# Analysis of Resonance Response Performance of C-Band Antenna Using Parasitic Element

**DOI:** 10.1155/2014/131374

**Published:** 2014-05-06

**Authors:** M. R. Zaman, M. T. Islam, N. Misran, J. S. Mandeep

**Affiliations:** ^1^Centre for Space Science (ANGKASA), Universiti Kebangsaan Malaysia (UKM), Bangi 43600, Malaysia; ^2^Department of Electrical, Electronic and Systems Engineering, Faculty of Engineering and Built Environment, Universiti Kebangsaan Malaysia (UKM), Bangi 43600, Malaysia

## Abstract

Analysis of the resonance response improvement of a planar C-band (4–8 GHz) antenna is proposed using parasitic element method. This parasitic element based method is validated for change in the active and parasitic antenna elements. A novel dual-band antenna for C-band application covering 5.7 GHz and 7.6 GHz is designed and fabricated. The antenna is composed of circular parasitic element with unequal microstrip lines at both sides and a rectangular partial ground plane. A fractional bandwidth of 13.5% has been achieved from 5.5 GHz to 6.3 GHz (WLAN band) for the lower band. The upper band covers from 7.1 GHz to 8 GHz with a fractional bandwidth of 12%. A gain of 6.4 dBi is achieved at the lower frequency and 4 dBi is achieved at the upper frequency. The VSWR of the antenna is less than 2 at the resonance frequency.

## 1. Introduction

It is a major concern for today's antenna designer to improve the antenna characteristics at given bands to work in. A lot of research is being conducted to make compact antennae for the ease of use. For compactness and light weight, microstrip antenna is becoming famous among researchers day by day all over the world [1]. As a new trend in microstrip antenna research, use of parasitic elements in antenna configuration has opened a new doorway within the field. The use of parasitic element is not explored much for C-band (4–8 GHz) [2–4]. A planar monopulse array antenna for C-band is shown in [5]. The antenna has a high array gain. Nonetheless, it is a multilayered antenna structure with an overall dimension of 210 × 210 mm^2^. A C-band antenna using electromagnetic band gap (EBG) structure is shown in [6]. This antenna consists of dual circular polarization. However, the antenna has a superstrate dimension of 365 × 365 mm^2^ which is bulky for C-band application. A diode controlled dual band antenna for C band is shown in [7]. The antenna uses a complex structure to connect the diode with the main body and attain dual band response. A band-notched antenna design method for UWB applications is shown in [8] that includes two parasitic elements at both sides of the active element to have band notch characteristic. Additionally the bottom layer has the same parasitic element with same dimensions. However, the gain of the antenna is comparatively adverse at the lower band. Another antenna is shown with four rectangular sized parasitic elements in [9]. The parasitic elements are required to achieve notch within the passband of the antenna. While optimizing using genetic algorithm, two parasitic elements were included to increase the bandwidth performance of the antenna in [10]. A slot is designed to act as a parasitic element for higher frequencies to increase the bandwidth and efficiency in [11]. By coupling with the monopole structure, the slot acts as a parasitic element. Nonetheless, the gain theta (*θ*) at the radiation pattern seems to increase more than gain phi (*φ*) when *φ* = 90° at higher frequencies. A bandwidth increment method is shown in [12] using proximity feeding technique. Among two antennas designed, one of the antennas has two square parasitic elements coupled with the proximity-fed antenna to increase the bandwidth. In spite of that, to increase antenna gain, the antenna has to compromise omnidirectional radiation pattern. A dual frequency antenna with two stacked parasitic element is shown in [13]. Although the parasitic elements assemble a bulky antenna, the antenna dual frequency bandwidth and gain are increased by controlling the coupling distance with the parasitic elements. In [14] a MIMO (Multi-Input Multi-Output) antenna is shown with parasitic element. Two parasitic elements are etched from the ground plane to achieve third mode resonance of the antenna. Nevertheless, the antenna gain is as low as 1.6 dBi for 5.2 GHz frequency. A multiband antenna with parasitic element is shown in [15]. The U-shaped parasitic element is responsible for the first and third mode resonance of the antenna. Despite that, the first mode resonance is narrower than other modes. The average gain and radiation efficiency has dropped at the first mode resonance. In [16] a microstrip fed dual band coplanar antenna is presented with frequency response at 2.4 GHz and 5.2 GHz without using any parasitic element. However, the gain of the antenna falls dramatically at the second resonance with an imbalanced radiation pattern. Using parasitic element, radiation performance enhancement is shown in [17]. The parasitic element shown in this paper gives a total radiation power improvement. Nonetheless, the antenna shown in this paper, without the parasitic element, the antenna bandwidth tends to degrade in personal communications service band. In [18], a multiband PIFA antenna design is shown with parasitic element at the ground plane that acts as a tuner at low frequencies and a parasitic radiator at high frequencies. A folded monopole UWB antenna is shown in [19]. An inverted “L” shaped element is used to increase the inductance at the capacitive folded monopole. Nevertheless, vertical radiation pattern caused by the parasitic element is more dominant in lower band compared to upper band causing omnidirectional radiation pattern at E-plane for the lower frequency. Dielectric resonator antenna (DRA) phased array is shown in [20] by using parasitic elements. Though the antenna has a bulky property, the antenna beam forming using parasitic dielectric loads is changeable manually. A wideband diversity antenna is designed using a parasitic element in [21]. The parasitic element is used to increase the isolation of the antenna where the element acts as a quarter wavelength open stub to suppress the surface current at the ground plane of the antenna for two closely situated radiating antennas. Nonetheless, the antenna efficiency is less than 80% for the operating bands. Mutual coupling reduction in MIMO antennas using parasitic monopoles is shown in [22]. The parasitic element is used to reduce the power transmission between two ports of the MIMO antenna. Despite that, by using the parasitic monopole, the antenna resonance response shifts inward.

We propose a novel dual band antenna design for C-band using parasitic elements in this paper. This parasitic element can create reverse coupling by reducing the mutual coupling between the active and parasitic elements. Two parasitic elements are introduced on the antenna patch. One of them is coupled with the feed line (only active element) of the antenna, and the other is coupled with the first parasitic element. It is to demonstrate the behavior of secondary parasitic coupling. The proposed antenna covers WLAN band at 5.8 GHz and upper frequency resonance at 7.6 GHz [23]. The proposed microstrip antenna is compact in size and square shaped with a dimension of 80 × 80 mm^2^. Current distributions are exhibited and studied to demonstrate the coupling technique. Finally, the proposed antenna is fabricated and measured to validate the findings.

## 2. Antenna Design

The proposed antenna is designed using FR4 substrate with a relative permittivity of 4.6 and a thickness of *h* = 1.6 mm. The effective relative permittivity is calculated using

(1) $$\begin{array}{c} \varepsilon_{e}=\frac{\varepsilon_{r}+1}{2}+\frac{\varepsilon_{r}-1}{2\left(\sqrt{12h/X+1}\right)}. \end{array}$$

**Figure 1:**
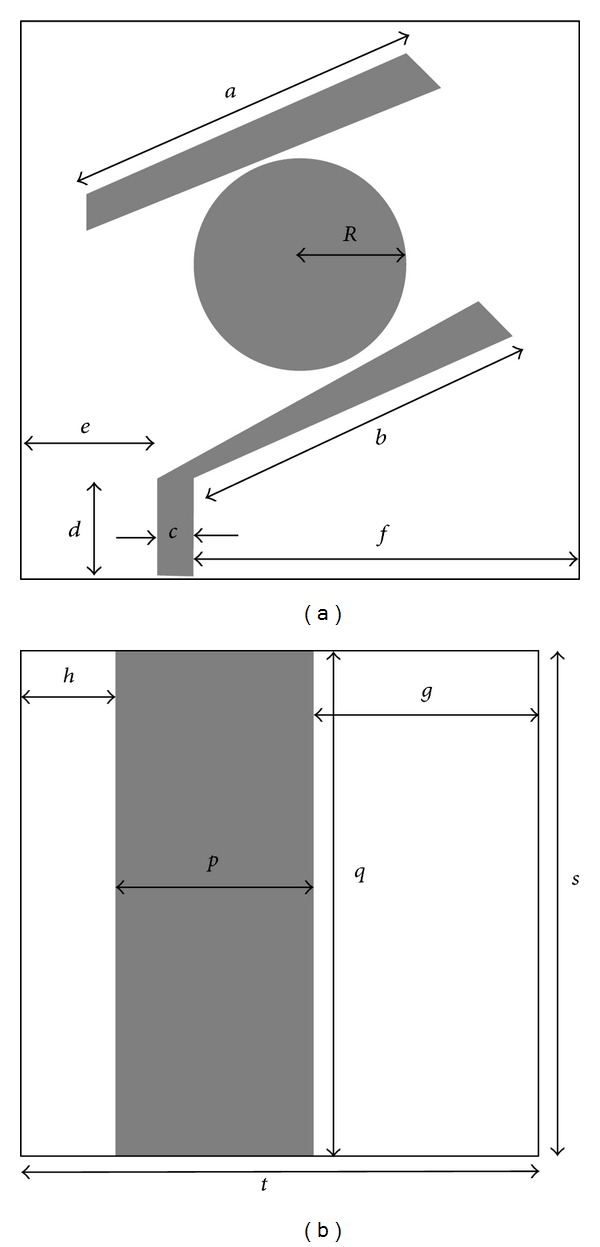
(a) Dimensions of the antenna patch and (b) dimensions of the antenna ground plane.

Here, *ε*_*r*_ = relative permittivity, *h* = substrate height, and *X* = strip width. The proposed antenna has a parasitic circle at the middle of the structure shown in Figure 1(a). A feed line of 15 mm length and 5 mm width is connected with a 50 Ω port along with a bended line of length 49.25 mm at the upper edge of the feed line. The bended line creates a coupling with the parasitic circle element. Another passive microstrip line is at the other side of the circle with a length of 50 mm. The ground plane shown in Figure 1(b) is rectangular shaped with the size of 80 mm in length and 30 mm in width. The total antenna dimension is 80 × 80 × 1.6 mm^3^. The height of the antenna is 1.6 mm; it is very small compared to the length and width of the antenna. The fields along *z* axis direction are quite unlikely to change with negligible height. The mode for the resonant frequencies TM_mn0_ can be found by using [24]

(2) $$\begin{array}{c} f_{\text{mn}0}=\frac{X_{\text{mn}}}{2\pi \times R_{e}\times \sqrt{\varepsilon_{e}}}\\ R_{e}=R\sqrt{\left[1+\frac{2\times h}{R\varepsilon_{e}\pi }\left\{\ln\left(\frac{R\pi }{2h}\right)+1.7726\right\}\right]}. \end{array}$$

Here, *X*_mn_ = zero of the derivative of the Bessel Function, *R*_*e*_ = effective radius of the patch, and *ε*_*e*_ = effective relative permittivity.

**Figure 2:**
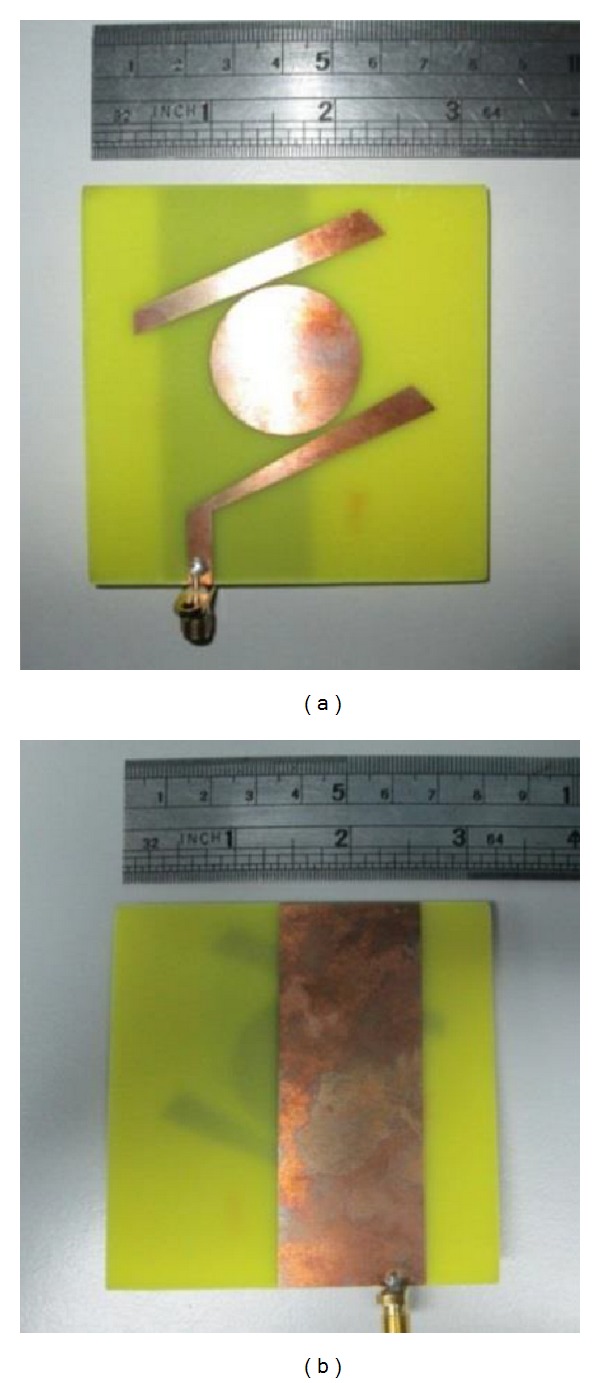
(a) Patch and (b) ground plane of the fabricated antenna prototype.

Figure 2 shows the fabricated antenna in FR4 substrate. The antenna is fed by a SMA coaxial connector with input impedance of 50 Ω. The dimensions of the antenna are shown in Table 2.

**Figure 3:**
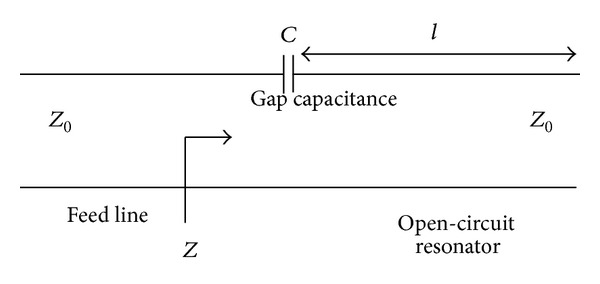
Equivalent circuit of the microstrip gap coupling.

Partial coupling is introduced using the microstrip line connected with the feed line. The coupling intensity is measured using the equation depended on the gap [25] between the parasitic circle and the tapered microstrip resonator. Using series capacitor, the equivalent circuit can be constructed as shown in Figure 3 for the gap in between microstrip lines. After ignoring the fringing fields the capacitor value can be calculated as

(3) $$\begin{array}{c} C=\frac{b_{c}}{\omega Z_{0}}, \end{array}$$

where *C* = coupling capacitor, *b*_*c*_ = normalized coupling capacitor susceptance, and *Z*_0_ = characteristics impedance. A negligible coupling grows between the two microstrip lines situated at the top and bottom side of the antenna. A sharp edge banding after the feed line is performed to have better resonance at the first resonance frequency.

## 3. Antenna Parameters Analysis

**Figure 4:**
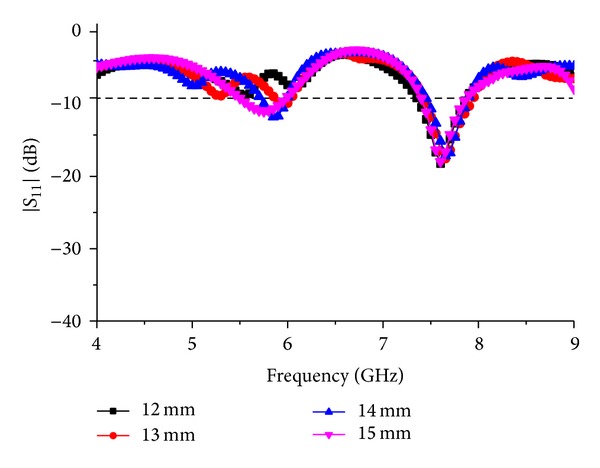
Response to the parametric change in the radius “*R*.”

To validate the proposed design and optimize the antenna structure, parametric analysis is performed. The major parts of the structure which are responsible for changing the S-parameter response of the antenna are modified to get the following parametric studies. This study will help to investigate the change in the impedance bandwidth along with the reflection coefficient. The main goal of this antenna is to achieve a coupling performance from two parasitic elements and analyze the coupling between them. The antenna is designed using FR4 substrate to achieve cost effectiveness and durability. With the use of RT/duroid substrate, the performance of the antenna can be enhanced; in this case decreasing the total fabrication cost is one of the main targets. The thickness of the FR4 substrate is preselected which is 1.6 mm. The thickness is chosen to make the antenna durable and applicable for outside applications.  Figure 4 shows the *S*_11_ response of the antenna for the parametric change in the radius “*R*” of the circle situated right in the middle of the antenna patch structure. The circle creates a coupling connection with the parasitic line above and the active line below. By changing the radius of the circle, the coupling effect between them changes and hence as a result the change in *S*_11_ can be seen in Figure 4. In this figure, a notch is introduced at 5.7 GHz, whereas the second resonance is still the same for the change in radius for *R* = 12 mm. The second resonance at 7.6 GHz shifts about 25 MHz below 7.6 GHz, where for the first resonance a notch is introduced at *R* = 13 mm. At *R* = 14 mm, the first resonance is shifted about 200 MHz above the desired resonance frequency. Also the first resonance response has become narrower, whereas the second resonance response stays the same. By using *R* = 15 mm, both of the resonance frequencies are found without any notch at the operating frequencies with a wide impedance bandwidth. From this graph, it can be concluded that, by changing the radius of the circle at the middle, the first resonance of the antenna can be tuned to have different operating regions.

**Figure 5:**
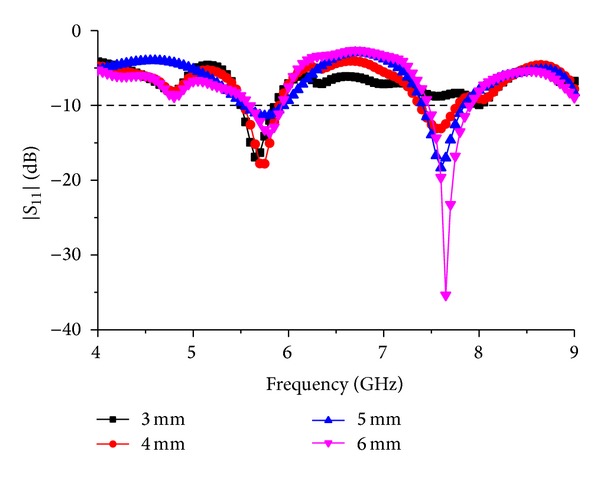
Response to the parametric change in feed-line width “*c*.”

Feed line is a crucial part of the design consideration for antennae operating at microwave frequencies. By changing only the feed line itself, antenna characteristics can be changed significantly. As the current propagation starts from the feed line and the other parts of the antenna depend on the flow of the current through the feed line, Figure 5 shows the response of the antenna for changes in the feed-line thickness “*c*.” For *c* = 3 mm, the antenna performance at first resonance gives response of lower than −15 dB with a −10 dB bandwidth starting from 5.5 GHz to 5.9 GHz. But this construction fully lacks the second resonance response. Again for *c* = 4 mm, the first resonance is almost as same as the resonance response for *c* = 3 mm. A −10 dB bandwidth starting from 7.4 GHz to 7.85 GHz can be seen for the second resonance response. At *c* = 5 mm, a little portion of the first resonance acts below −10 dB which is acceptable as a resonance frequency for the antenna. Furthermore, compared to the other constructions, the resonance is wide and physically it will be easier to achieve. For the second resonance, the setup shows a stable and wide bandwidth performance of 0.4 GHz. For *c* = 6 mm, the antenna shows a narrow bandwidth performance at the first resonance starting from 5.55 GHz till 5.95 GHz. The second resonance response is as low as −35.4 dB at 7.65 GHz with a bandwidth starting from 7.45 GH to 7.95 GHz.

**Figure 6:**
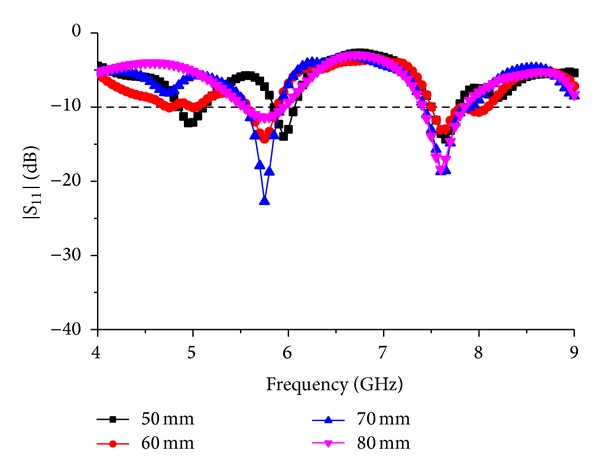
Response to the parametric change in ground plane length “*q*.”

By changing the length and width of the partial ground plane, different response can be found.  Figure 6 shows the response to the parametric change in the ground plane length “*q*.” The study is carried out using large steps to find major changes in the resonance response of the antenna. It can be observed that for *q* = 50 mm a total number of 3 resonances can be found in the *S*_11_ response. The resonance frequencies are at 4.95 GHz, 5.95 GHz, and 7.65 GHz with a bandwidth of 0.3 GHz, 0.2 GHz, and 0.3 GHz, respectively. With *q* = 60 mm, the antenna shows a narrow bandwidth at the first resonance and comparatively wider bandwidth at the second resonance. The resonance frequencies are at 5.75 GHz and 7.65 GHz with a bandwidth of 0.1 GHz and 0.6 GHz, respectively. Also the response shows a wideband notch in the middle of two resonances. For *q* = 70 mm the antenna resonance response is limited to two frequencies where the first resonance response is at 5.75 GHz with *S*_11_ response of less than −22 dB and the second resonance response is at 7.6 GHz with a reflection coefficient of less than −18 dB. For *q* = 80 mm, the antenna gives a wide bandwidth of 0.8 GHz at 5.7 GHz resonance frequency and 0.4 GHz at 7.6 GHz resonance frequency.

**Figure 7:**
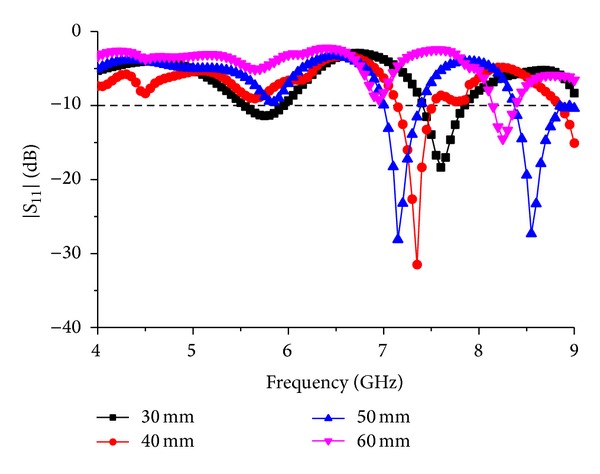
Response to the parametric change in the ground plane width “*p*”.

Figure 7 shows the response to the parametric change in the ground plane width “*p*.” A step of 10 mm is taken between 30 mm and 60 mm for the parametric studies. For *p* = 60 mm, the antenna frequency response alters dramatically. First resonance frequency cannot be seen for this setup of the antenna, whereas the second frequency shifts about 0.5 GHz from the optimum resonance response of the antenna with a narrow band of 0.25 GHz. First resonance of the antenna becomes very narrow when *p* = 50 mm with resonance response above −10 dB. There are two more resonance frequencies situated at 7.15 GHz and 8.55 GHz with a reflection coefficient of −28 dB and −27 dB, respectively. The bandwidths are 0.4 GHz and 0.6 GHz, respectively. For *p* = 40 mm, the first resonance is amended fully, whereas the second resonance is shifted inwards with a reflection coefficient of −31 dB at 7.35 GHz. It can be observed that, for *p* = 30 mm, we find the optimum resonance response with a bandwidth of 0.8 GHz at 5.7 GHz resonance frequency and 0.4 GHz at 7.6 GHz resonance frequency.

## 4. Results and Discussion

The proposed antenna is measured using Agilent E8362C power network analyzer. The design and simulation of the antenna are carried out using commercially available software HFSS (High Frequency Structure Simulator) by Ansys Corporation. This software uses finite element method (FEM) to calculate the antenna radiation characteristics and the resonance frequency.

**Figure 8:**
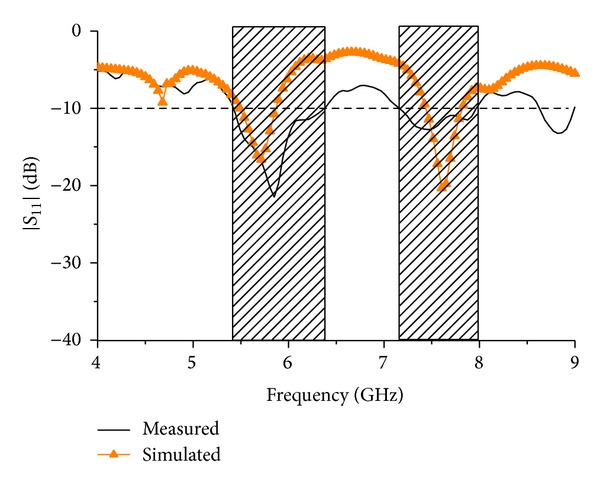
*S*_11_ response of the antenna.

Figure 8 shows the simulated and measured *S*_11_ response of the proposed antenna after optimization wielding the parametric studies above. It can be observed that the simulated result and the measured result tend to agree with each other. However, the measured result shows increased bandwidth at the resonance frequencies compared to the simulated result. At the first resonance the bandwidth is 0.9 GHz starting from 5.4 GHz till 6.3 GHz which is 0.1 GHz increment from the simulated result. Again at the second resonance, the bandwidth of the measured result is 0.9 GHz starting from 7.1 GHz till 8.0 GHz which is 0.5 GHz increment from the simulated resonance response. Although the measured result shows wider bandwidth, nonetheless both of resonance responses are almost at the same frequencies. Although the first mode resonance behaves as depicted in the *S*_11_ parameter simulation, the second mode resonance bandwidth is increased and the resonance point is shifted from the simulation. This could be due to the higher frequency current leakage by the connecting wire.

**Figure 9:**
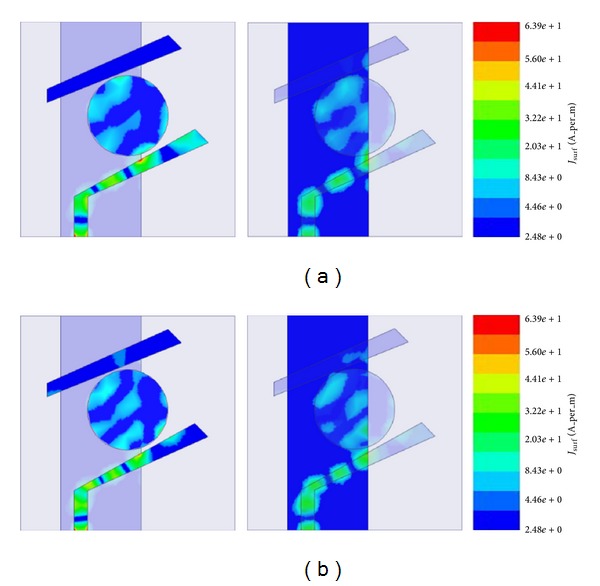
Surface current distribution of patch and ground plane at (a) 5.7 GHz and (b) 7.6 GHz in Amp/m.

Figure 9 shows the surface current distribution of the antenna at two resonance frequencies. It can be seen for both resonance frequencies that the edge of the active microstrip line close to the circle is showing the most intensive current distribution which amounts 64 Am^−1^ for 5.7 GHz and 65.5 Am^−1^ for 7.6 GHz. It is due to the coupling between the circle and feed line. The outer microstrip patch with length “*a*” does not have significant current passing inside of it compared to others, because it is the second parasitic element and stays further from the feed line compared to the parasitic circle. Moreover from the current distribution pattern at the active element, it can be seen that, there are nulls introduced periodically which can be due to the coupling with the parasitic circle. Considering the current distribution at the ground plane, the same periodical null pattern can be found for both resonance frequencies. The ground plane shows a very slim connection with the secondary parasitic element at the upper edge of the antenna. Again for both frequencies, it can be seen that there is a surrounded current distribution pattern within the edges of the parasitic circle, which shows that the first coupling is strong enough to distribute the active elements current to the first passive element.

**Figure 10:**
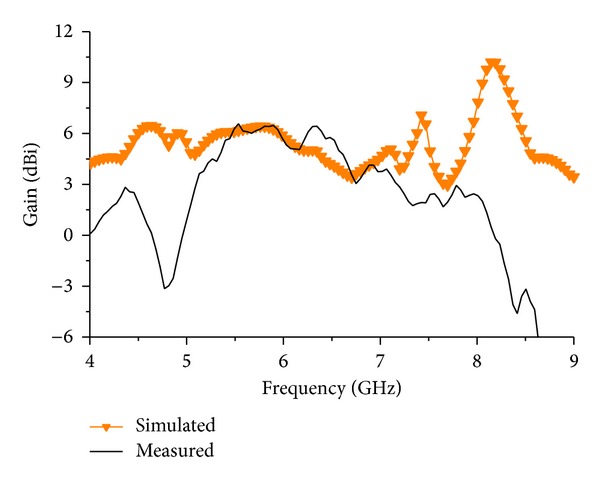
Gain of the proposed microstrip antenna.

**Figure 11:**
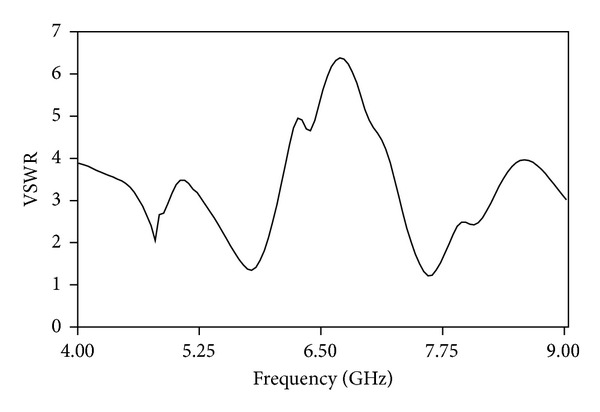
VSWR of the proposed microstrip antenna.

Figure 10 shows the simulated and measured gain of the antenna. It can be observed that the average simulated gain is bigger than the average measured gain. Moreover, at the WLAN operating frequency the measured gain stays almost the same as the simulated gain which is 6.16 dBi. For the second resonance mode, the measured gain is 2.5 dBi which is about 1.6 dBi less than the simulated gain. The measured gain can be affected by the geometry achieved for the antenna. As the antenna characteristics depend more on the coupling between the patch elements, improper coupling can reduce the total gain of the antenna. A fractional bandwidth of 13.5% is measured at the first resonance frequency and a fractional bandwidth of 12% is measured at the second resonance frequency. The VSWR result is shown in Figure 11. It shows, at the resonance frequencies, the VSWR is less than 2 at an average. This shows that the loss due to wave reflection is minimized at the antenna operating region. The proposed antenna characteristics are tabulated in Table 1.

**Figure 12:**
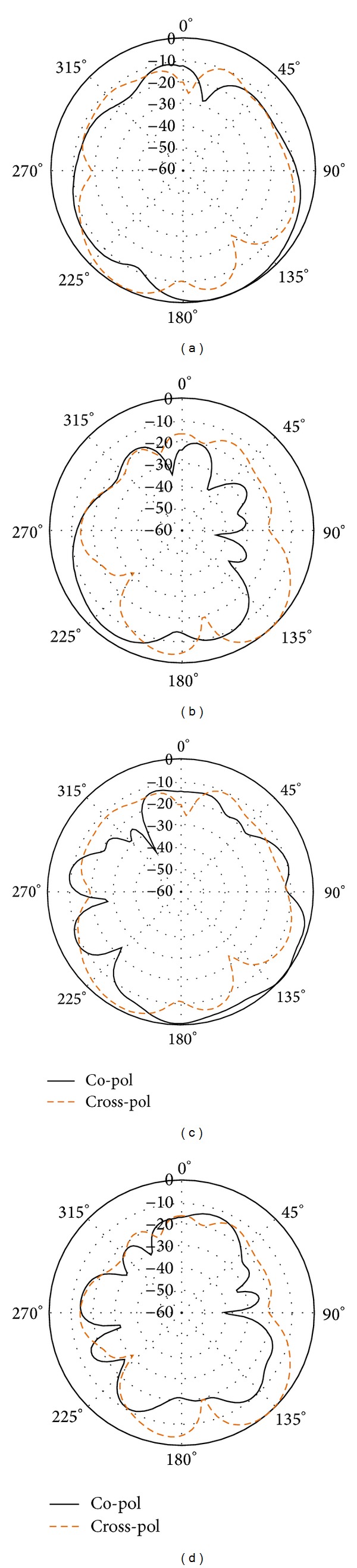
Simulated normalized radiation pattern of the antenna at (a) 5.7 GHz E-plane, (b) 5.7 GHz H-plane, (c) 7.6 GHz E-plane, and (d) 7.6 GHz H-plane.

**Figure 13:**
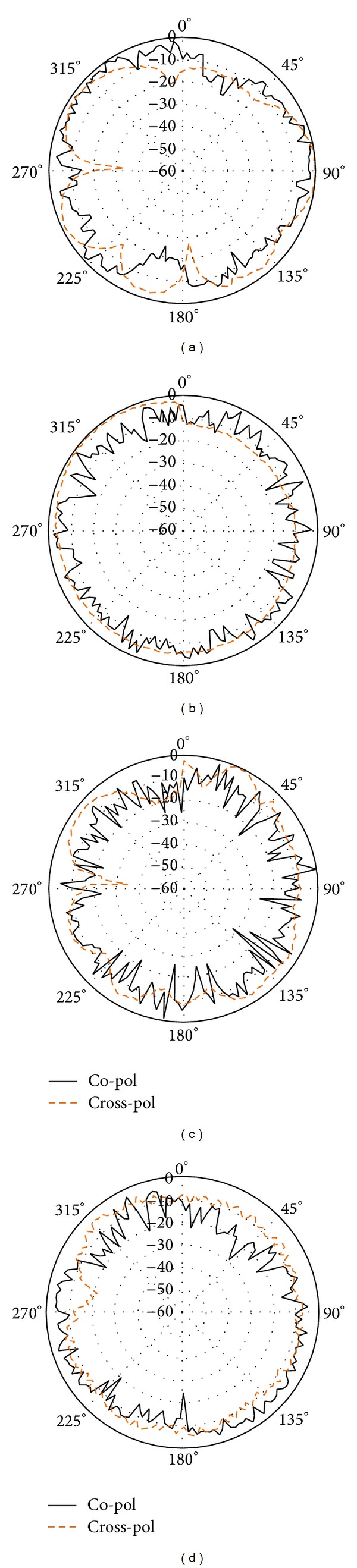
Measured normalized radiation pattern of the antenna at (a) 5.7 GHz E-plane, (b) 5.7 GHz H-plane, (c) 7.6 GHz E-plane, and (d) 7.6 GHz H-plane.

Figures 12 and 13 show the simulated and measured normalized radiation pattern of the antenna, respectively. It can be observed that, in Figures 12(a), 12(b), and 12(c), the co-pol beam of the antenna shows direction properties. However, Figure 12(d) does not follow the same pattern. In Figure 13, we can observe noise is introduced in the co- and cross-pol radiation pattern. The noise is due to the long wire from the VNA till the turn table inside anechoic chamber. As the default minimum scale is set to −60 dB, most of the direction pattern is not observed.

Calibration is performed before measurement. Nonetheless, the difference between the measured and simulated result took place due to the fact that shape of the parasitic element is not achieved exactly as in the simulation. Also the length of the cable used in the measurement shoot leakage current out of its radial plane at higher frequencies.

**Figure 14:**
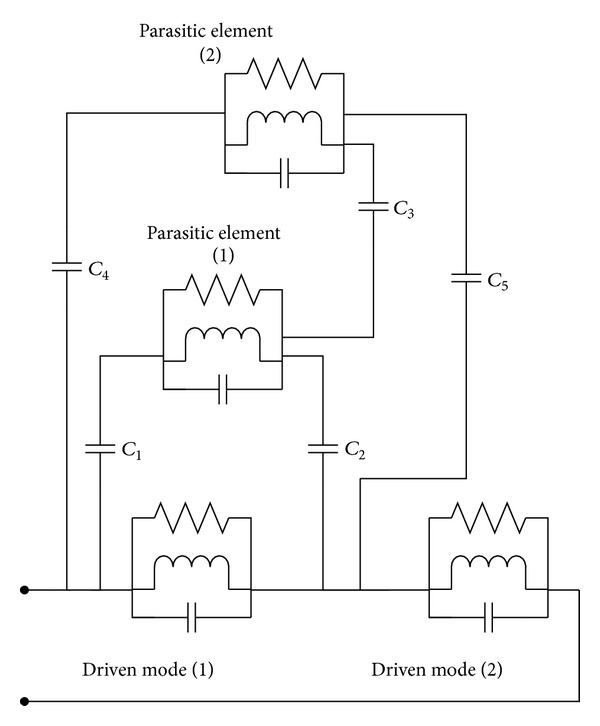
Electrical model of the parasitic element dual-mode C-band antenna.

Figure 14 shows the electrical model of the proposed antenna. The driven modes 1 and 2 RLC circuits represent the patch at two-resonance mode of the antenna. Parasitic elements 1 and 2 RLC circuits depict the two parasitic element of the antenna. The coupling between two parasitic elements is contemplated using *C*_3_. Driven mode 1 is coupled with the parasitic element 1 constituted by *C*_1_ and driven mode 2 is coupled with the parasitic element 1 represented by *C*_2_. *C*_4_ and *C*_5_ represent the coupling between parasitic element 2 and driven modes 1 and 2, respectively. However, the current distribution pattern shows the coupling between the parasitic element 2 and driven modes 1 and 2 are negligible.

## 5. Conclusion

Use of parasitic element for obtaining dual mode response is shown in this paper. A novel dual band microstrip antenna for C-band application is presented. The antenna shows coupling response using a circular parasitic element partially coupled with the active feed line. A secondary coupling is shown in this paper using secondary parasitic element and primary parasitic element. A relation between two parasitic elements is shown using the current distribution pattern at the resonances of the antenna. The proposed antenna achieves a −10 dB impedance bandwidth of 800 MHz (from 5.5 GHz to 6.3 GHz) of impedance bandwidth at the lower frequency (WLAN) and 900 MHz (from 7.1 to 8 GHz) of impedance bandwidth at the upper frequency. The measured gains are 6.16 dBi and 2.5 dBi at 5.7 GHz and 7.6 GHz, respectively.

## Figures and Tables

**Table 1:** Performance of the antenna at resonance frequencies.

First resonance	
Frequency (GHz)	5.7
Bandwidth (GHz)	0.8
Gain (dBi)	6.4
Second resonance	
Frequency (GHz)	7.6
Bandwidth (GHz)	0.9
Gain (dBi)	4.04

**Table 2:** Design parameters of the proposed antenna.

Dimension	Value (mm)
Length, *a*	50
Length, *b*	49.25
Feed width, *c*	5
Feed line, *d*	15
Gap, *e*	20
Gap, *f*	55
Radius, *R*	15
Gap, *g*	35
Gap, *h*	15
Width, *p*	30
Length, *q*	80
Antenna length, *s*	80
Antenna width, *t*	80
Substrate thickness	1.6
